# Collagen and Its Derivatives Serving Biomedical Purposes: A Review

**DOI:** 10.3390/polym16182668

**Published:** 2024-09-22

**Authors:** Hanna Wosicka-Frąckowiak, Kornelia Poniedziałek, Stanisław Woźny, Mateusz Kuprianowicz, Martyna Nyga, Barbara Jadach, Bartłomiej Milanowski

**Affiliations:** 1 GENERICA Pharmaceutical Lab, Regionalne Centrum Zdrowia Sp. z o.o., ul. Na Kępie 3, 64-360 Zbąszyń, Poland; h.wosicka-frackowiak@rcz-zbaszyn.pl (H.W.-F.); k.poniedzialek@rcz-zbaszyn.pl (K.P.); s.wozny@rcz-zbaszyn.pl (S.W.); m.kuprianowicz@rcz-zbaszyn.pl (M.K.); m.nyga@rcz-zbaszyn.pl (M.N.); 2 Chair and Department of Pharmaceutical Technology, Faculty of Pharmacy, Poznan University of Medical Sciences, ul. Rokietnicka 3, 60-806 Poznan, Poland; bajadach@ump.edu.pl

**Keywords:** biomaterial, biocompatibility, collagen, derivatives of collagen, medical applications, tissue engineering and regeneration, drug delivery systems, bioinks for 3D bioprinting

## Abstract

Biomaterials have been the subject of extensive research, and their applications in medicine and pharmacy are expanding rapidly. Collagen and its derivatives stand out as valuable biomaterials due to their high biocompatibility, biodegradability, and lack of toxicity and immunogenicity. This review comprehensively examines collagen from various sources, its extraction and processing methods, and its structural and functional properties. Preserving the native state of collagen is crucial for maintaining its beneficial characteristics. The challenges associated with chemically modifying collagen to tailor its properties for specific clinical needs are also addressed. The review discusses various collagen-based biomaterials, including solutions, hydrogels, powders, sponges, scaffolds, and thin films. These materials have broad applications in regenerative medicine, tissue engineering, drug delivery, and wound healing. Additionally, the review highlights current research trends related to collagen and its derivatives. These trends may significantly influence future developments, such as using collagen-based bioinks for 3D bioprinting or exploring new collagen nanoparticle preparation methods and drug delivery systems.

## 1. Introduction

Since ancient times, humans have sought ways to accelerate wound healing and restore lost bodily functions. Modern medicine, driven by increasing clinical demands, has rapidly developed advanced materials with potential therapeutic applications [1].

While the definition of a biomaterial evolves with scientific progress, the most commonly accepted one is proposed by the European Society for Biomaterials Consensus Conference II: “A biomaterial is a material intended to interface with biological systems to evaluate, treat, augment or replace any tissue, organ or function of the body” [2,3]. The prefix bio- emphasizes the material’s compatibility with human tissues and the body’s response rather than indicating a biological origin [4].

For a material to be considered a biomaterial, it must meet several criteria. These include biocompatibility (the ability to coexist with body tissues without causing harmful reactions), biofunctionality, bioinertness, and bioactivity. Additionally, the biomaterial should be sterilizable and must not induce inflammatory, toxic, or allergic reactions [5].

Biomaterials can be natural (e.g., collagen, chitin) or synthetic. Based on material properties, they can be divided into metallic (e.g., titanium, stainless steel), ceramic (e.g., zirconia, hydroxyapatite), polymeric (e.g., silicon, nylon), and composite materials (combining the properties of various materials, e.g., carbon fibers, ceramic particles) [3,6]. Polymeric materials are gaining popularity due to their diverse properties and ease of processing. The global medical polymer market is around USD 1 billion [7].

Due to their specific characteristics, biomaterials are primarily used for medical purposes. The applications in medicine are extensive and include orthopedics (e.g., implants such as an artificial hip or artificial knee), ophthalmology (e.g., intraocular lenses), cardiology (e.g., heart valve), dentistry, plastic surgery, urology, drug delivery, and many other fields [4,8]. Thanks to ongoing research and the development of new materials, even more significant advances in this field can be expected.

One of the most widespread and studied natural biomaterials is collagen (CLG). Its biocompatibility, biodegradability, and common availability make it an attractive material for many biomedical applications. The diverse types of CLG, differing in structure and properties, enable its use in various applications [9,10].

Collagen’s ability to self-organize and form three-dimensional fiber networks through cross-linking provides appropriate mechanical strength, making it an excellent material for constructing tissue scaffolds [10,11]. Moreover, CLG may interact with cell surface receptors, enabling strong cell adhesion, proliferation, and migration, which are crucial for healing and tissue regeneration [12]. Through the chemical modifications of CLG, its properties can be precisely adjusted to specific needs. For example, introducing functional groups can increase hydrophilicity, bioactivity, or the ability to form bonds with other materials [13].

Although many researchers have explored the issue of CLG application for medical purposes [14,15,16,17,18,19,20], most of them have omitted the significant role of the protein’s nativeness on the characteristics and effectiveness of the final biomaterial. Hence, this review presents a detailed description of the structure of CLG and its fundamental properties that determine its wide use in regenerative medicine. Various CLG-derived biomaterials and their applications in medicine have been elaborately discussed. Furthermore, the common research trends are presented to highlight the emerging applications of CLG.

## 2. Characteristics of CLG

### 2.1. Structure and Properties

**Figure 1:**
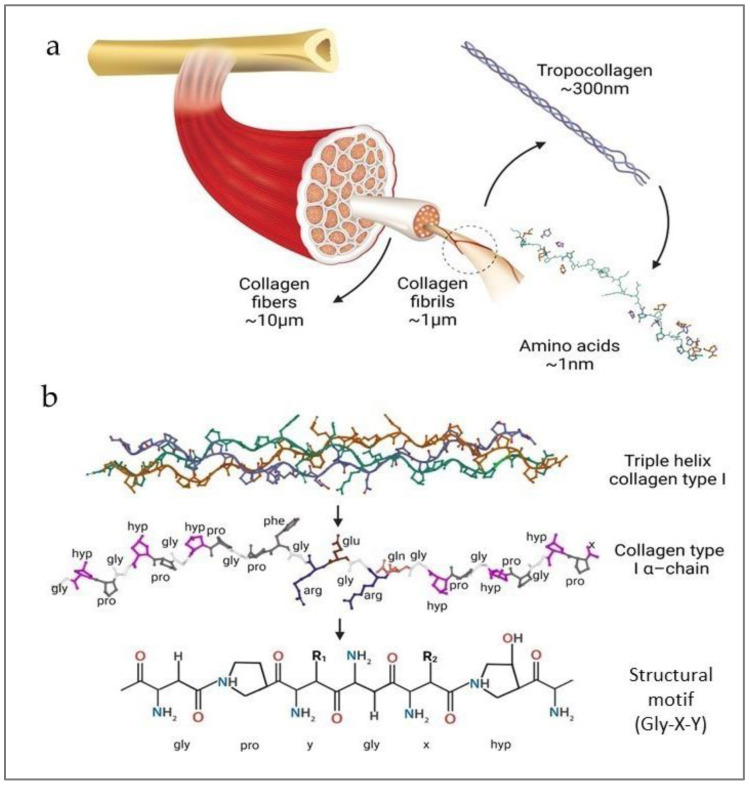
(**a**) Structure of CLG fibers; (**b**) molecular structures of the triple helix (reprinted from [16]).

CLG is an extracellular structural protein, constituting almost one-third of the total protein mass in the human body. As the most important component of the extracellular skin matrix, it is responsible for its elasticity and durability [21,22]. CLG exhibits a hierarchical structure, from its primary amino acid sequence to its organization into complex fibers. Also, CLG belongs to the group of fibrillar proteins responsible for the structure and biochemical properties of connective tissue in mammals. There are many types of CLG (28 types, numbered with Roman numerals), and they are the most complex natural polymers [22,23,24]. The structure of these protein molecules is based on more than 20 amino acids, and the most abundant are proline (Pro), hydroxyproline (Pro-OH), glycine (Gly), and hydroxylysine (Lys-OH) [21,25]. Its molecular composition is fundamental to its structural integrity and function. The CLG structure is based on the superhelix, which is held together by hydrogen bonds, electrostatic interactions, and van der Waals forces [26,27]. Also, Gly, the smallest amino acid, appears at every third position in the sequence, allowing the close packing necessary for the triple helix formation. Pro and Pro-OH play crucial roles in stabilizing the helical structure through their ring structures, which impose conformational constraints [21,25]. The unique structure of CLG consists of three left-handed polypeptide α chains, the so-called procollagen (Figure 1). They are wrapped around each other and have a common axis, thus forming a right-handed superhelix conformation called tropocollagen [21,25].

The primary structure of CLG refers to the specific sequence of amino acids in CLG polypeptide chains. It is characterized by repeating Gly-X-Y triplets, where X and Y are often PRO and PRO-OH, respectively (Figure 1b). Furthermore, the triple helix is stabilized thanks to the Gly, high Pro content, and Pro-OH forming hydrogen bonds and electrostatic interactions between lysine and aspartic acid [14,21,22]. The unique structure of the triple helix and the strong bonds between amino acids help the CLG fibers maintain their flexibility and resistance to stretching. The triple helix is 300 nm long and 1.5 nm in diameter [16,28]. Telopeptides are responsible for the protein’s immunogenicity on both structure ends. The α chains range from 662 (human α 1(X)) to as many as 3152 (human α 3(VI)) amino acids in length [23]. The triple helix chains can be identical or different and form homotrimers (CLG types II, III, VII, VIII, and X) or heterotrimers (CLG types I, IV, V, VI, IX, and XI), respectively. CLG fibrils (Figure 1a) are formed by the staggered arrangement of CLG molecules [14,16], creating a repeating banding pattern visible under electron microscopy. Furthermore, CLG fibrils aggregate to form larger fibers. These fibers contribute significantly to the mechanical properties of tissues, providing tensile strength and flexibility [16,27]. Covalent cross-links are formed between lysine (Lys) or hydroxylysine (Lys-OH) amino acids, further stabilizing the fibrils. The extent of cross-linking increases with age, enhancing the tensile strength of the CLG fibers but also making them more rigid and less elastic [29,30]. There are at least 28 different types of CLG [16,21,22], each serving distinct functions, and they are present in various tissues. Type I CLG is the most abundant in skin, tendon, and bone. Type II CLG is predominant in cartilage, where it resists compressive forces. Type III CLG is often found alongside Type I, particularly in the skin, blood vessels, and internal organs, contributing to tissue elasticity and repair. Other types of CLG, such as Type IV and Type V, play specialized roles in basement membranes and cell surfaces, respectively [21,22,23,31]. The elasticity of CLG refers to its ability to return to its original shape after being stretched or deformed [22,31]. This critical property allows various tissues to withstand mechanical stress and strain and is essential for tissues that require flexibility and the ability to absorb mechanical forces without sustaining damage [21,22].

The triple helical structure of CLG fibers contributes to its tensile strength and elasticity. The helical shape allows the fibers to stretch and return to their original form [16,22]. Also, covalent bonds, or cross-links, between CLG molecules, as mentioned above, provide structural integrity and enhance the elastic properties by preventing the fibers from sliding past each other too easily [29,30]. CLG exhibits a non-linear stress–strain curve. Initially, there is a low-stiffness region (toe region) where the crimped fibrils straighten. This is followed by a linear region where the CLG fibers are directly stretched. It also displays viscoelastic properties, meaning it has viscous and elastic characteristics. This dissipates energy and provides damping, which is essential for tissues undergoing cyclic loading [32]. Age, disease, and environmental exposure can affect collagen’s elasticity, impacting tissue function and integrity. With aging, CLG undergoes changes such as increased cross-linking and glycation, reducing elasticity and making tissues stiffer [33,34]. Certain diseases, like osteoarthritis, can affect the quality and organization of CLG, leading to reduced elasticity and compromised function of the affected tissues [35]. UV radiation and environmental factors can degrade CLG, leading to a loss of elasticity, particularly in the skin [30,36].

### 2.2. Types of CLG and Their Origin

CLG can be sourced from various animals, and the most common sources [37,38,39,40,41,42] include pigs (porcine collagen), cows (bovine collagen), and fish (marine collagen). However, attempts have also been made to obtain it from sheepskin [43]. Porcine CLG is usually derived from porcine skin, bones, and connective tissues, while bovine collagen sources are skin, bones, and cartilage. Both types of CLG (porcine and bovine) are rich in Gly, Pro, and Pro-OH, which are essential for CLG synthesis and structure. Porcine CLG has high compatibility with human CLG due to structural similarity, making it useful for medical and cosmetic applications [38,40]. Bovine CLG is generally well tolerated, though some individuals may have sensitivities or dietary restrictions related to bovine products. In medicine, pork CLG is widely used in wound dressings, skin grafts, and surgical sutures due to its biocompatibility and ability to promote healing [44]. In comparison, bovine CLG is utilized in tissue scaffolding, bone grafts, and joint health supplements [45]. Regarding cosmetics, porcine and bovine CLG are incorporated into anti-aging products, moisturizers, and serums to improve skin elasticity and hydration [34,46,47]. In dietary supplements, CLGs are available in powder form or capsules to support joint health and skin, hair, and nail strength [48]. As far as the food industry is concerned, CLG derivatives are used mainly in the form of gelatin as food additives in the production of desserts, gummy candies, and other food products. Marine CLG is received from fish skin, scales, bones, and fins, especially from cold-water species like cod, salmon, and pollock [22,49]. This type of CLG contains a high concentration of amino acids (Gly, Pro, and Pro-OH). However, it also has smaller peptide molecules, which leads to better bioavailability [49,50]. Smaller peptide size often makes it more effective for quick absorption and utilization in the body. Marine CLG has a higher absorption rate than bovine and porcine CLG, making it highly effective in supplements and skincare products. It is also considered more eco-friendly as fish by-products are used that would otherwise be discarded. Marine CLG is less common in the food industry than bovine and porcine gelatin, but it is used in some niche products targeting health-conscious consumers. In addition, it is also often chosen by those with sensitivities to mammalian CLG sources, though fish allergies must be considered.

### 2.3. Methods of Obtaining

**Figure 2:**
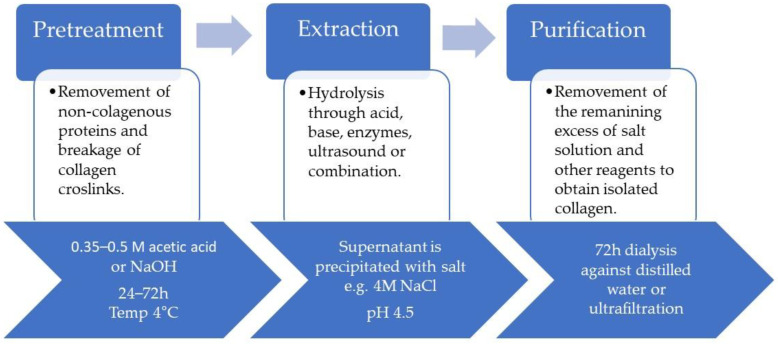
Steps of CLG isolation process (based on [16]).

To obtain CLG, extraction and hydrolysis are needed. The extraction process depends on the source material; for example, enzymes or acids must hydrolyze extracted proteins from pigs or cows [51,52]. Hydrolysis is commonly used to break the CLG into gelatin or hydrolyzed CLG peptides. Purification follows hydrolysis, and the CLG is purified to remove non-collagenous proteins and other impurities [37,38,39,40]. Marine CLG is hydrolyzed to produce smaller peptides that are more easily absorbed by the body. Also, extensive purification processes ensure that the received CLG is free from allergens and impurities [41,49,53]. The recovery process typically involves three steps: pretreatment of the source tissue, CLG extraction, and purification (Figure 2). The extraction temperature is controlled; a low value (4 °C) minimizes CLG degradation.

The pretreatment stages are usually preceded by soaking the skin in cold water for a few days and washing it by changing the water every few hours [16]. The skin is then cut into pieces that are easier to process. During the pretreatment stage, the covalent intermolecular cross-links between the CLG molecules are broken. This process is prolonged, even in boiling water; so, various mild chemical treatments are necessary. Diluted acids and alkalis are typically used for partial hydrolysis of the CLG [54,55]. For acid pretreatment, washed and chopped skin pieces are immersed in dilute acid at a controlled temperature. Acid pretreatment is suitable for relatively fragile skins [16] with a lower degree of fiber intertwinement, such as porcine and fish skins. Dilute alkalis, such as sodium hydroxide (NaOH) and calcium hydroxide (Ca(OH)_2_), are used for pretreatment, and the duration of this step depends on the thickness of the material being treated. Alkalis are particularly effective in extracting CLG from thick and hard materials and hydrolyzing undesirable non-collagenous proteins, lipids, pigments, and other organic materials [56]. The temperature, duration, and concentration of the alkali significantly influence the effectiveness of the removal of unwanted non-collagen materials [42]. The conventional extraction of CLG from different sources is typically based on chemical hydrolysis using acid, alkali, or salt solubilization. Organic acids, such as acetic, chloroacetic, citric, or lactic acids, are used. Inorganic acids (hydrochloric, sulfuric, and nitric) can also be used [16]. Under acidic conditions, CLG molecules have a net positive charge, and the resulting electrostatic repulsive force between them facilitates molecular separation. During acid-based extraction, raw material, such as porcine or fish skin, is soaked in 0.5 M acetic acid at a controlled temperature until swelling, usually between 24 and 72 h, followed by precipitation [16,55]. In the next step, the precipitate is centrifuged, and the supernatant is then salted out for 12 h with NaCl [42,56]. The supernatant is re-dissolved in 0.5 M of acetic acid and then dialyzed for a few days against cold distilled water, lyophilized, and stored until further use [54]. Sodium hydroxide or potassium hydroxide are the most common bases used. However, calcium oxide, calcium hydroxide, or sodium carbonate can also be used as extractants [42,57], and the process can take several days to several weeks [56]. Instead, this method is used for thick materials, such as leather waste or bovine shavings, because it penetrates more aggressively to swell the material [16,54]. Usually, for soaking, 0.05–0.1 M NaOH is used. The received CLG is then precipitated by dialysis in 50 mM Tris-HCl buffer (pH 7.4), and the collected precipitate is dissolved in 5 mM acetic acid [42]. Organic acids are more effective at cleaving CLG cross-links, resulting in higher extraction efficiency [57,58] than mineral acids, and can solubilize non-cross-linked CLG. While alkalis tend to hydrolyze CLG fibrils, amino acids, like cysteine, histidine, serine, and threonine, may be destroyed [42,52]. Sometimes, ultrasounds, microwaves, and enzymes are added to the method. Pepsin, tryptase, and papain [56] are the most common enzymes used for CLG extraction [16]. They relax cross-linking in CLG by cleaving aminotelopeptides from the tropocollagen molecule. This method is most useful in adult tissue, where strong intermolecular bonds are formed due to ketoimine cross-links [42,59]. In enzymatic hydrolysis, the raw material or residue is soaked in 0.5 M acetic acid or 0.01 M HCl containing selected enzymes, such as 0.1 percent (*w*/*v*) pepsin. The mixture is continuously stirred at 4 °C for approximately 48 h. After this time, the solution is filtered or purified, and the supernatant is salted out to precipitate. For all the processes, the last step involves separation by centrifugation, re-dissolution in acid, and dialysis to obtain acid-soluble CLG [42]. The extraction methods can be adjusted depending on the final product’s properties and the process’s effectiveness [42,53]. The features of the final product, such as the average length of the polypeptide chains, solubility, viscosity, thermal stability, emulsifying capacity, and water retention, are influenced by the specifics of the extraction method. 

### 2.4. Native CLG vs. Cross-Linked CLG

Native and cross-linked CLG represent two different forms used in various biomedical applications. Native CLG means a natural, unmodified form, with a preserved triple helix structure, which is crucial for its biological function and mechanical properties [44,60]. Some researchers claim that to provide an optimal environment for cellular infiltration, morphology, and viability, the nativeness of CLG in a biomaterial and its cellular binding sites need to be retained [61]. It has to be emphasized that to preserve all these native properties, the processes of CLG preparation and purification have to be carefully chosen. Cross-linked CLG, on the other hand, undergoes chemical or physical treatment to introduce covalent bonds between its molecules [62,63,64]. This process enhances its stability and mechanical properties. It can be obtained by agents such as glutaraldehyde, carbodiimides, or genipin (chemical cross-linking) or by the use of UV irradiation or dehydrothermal treatment (physical cross-linking) [63,64]. The cross-linking process enhances CLG resistance to enzymatic and thermal degradation compared to native CLG [65]. Cross-linked CLG possesses greater tensile strength and durability, which makes it suitable for load-bearing applications [14,62,66]. What is also exciting is that cross-linking can be adjusted to control the degradation rate for applications requiring long-term stability. Rasouli et al. have provided helpful guidance for CLG cross-linking methods. UVC, as a physical cross-linking method, preserves the integrin binding sites on CLG, resulting in a dose-dependent improvement in mechanical performance up to a dose of 5 J cm^−2^. At higher doses, it leads to CLG fragmentation. Genipin, a non-cytotoxic plant-based cross-linker, results in moderate improvements in mechanical performance compared to UVC. EDC-NHS (carbodiimide and N-hydroxysuccinimide) also results in modest improvements, with the possibility of losing some integrin binding sites on the CLG. Glutaraldehyde dramatically improves the ultimate tensile strength compared to human and rat tendons, preserving the native CLG molecular structure. However, cytotoxicity is an inherent disadvantage of using glutaraldehyde as a cross-linker [65]. Native CLG has superior biocompatibility and bioactivity due to the preservation of natural binding sites and minimal modifications, and that is why it deserves significant attention, which it very often lacks [44,60].

The functionality of native CLG becomes obvious in clotting experiments. It is assumed that a fast and reliable triggering of platelet aggregation indicates that the quaternary structure of CLG is intact. Wiegand et al. [67] compared three different CLG wound dressings in vitro. During the evaluation of platelet aggregation, only the dressing made from native CLG showed an activation of the clotting cascade. Fibroblasts seeded on the native CLG showed exponential growth over 14 days, whereas those seeded on the other two dressings exhibited meager proliferation rates. It has been shown that native CLG allows more efficient angiogenesis and more significant fibroblast chemotaxis than denatured CLG in vitro. In intensely processed or degraded CLGs, the bioactive potential of cells involved in wound healing is likely to be lost or attenuated [67]. Native CLG also shows a faster degradation rate due to natural enzymatic breakdown, which can be advantageous for applications requiring rapid tissue regeneration. Some authors even show that the importance of the manufacturing process and the ability to save the nativeness of CLG is more critical for the performance of CLG matrices than the animal source of CLG [68]. Cross-linked CLG can sometimes show reduced bioactivity and slower, controlled degradation, and that is why it can be suitable for long-term applications where prolonged structural support is needed. Good mechanical properties make it suitable for load-bearing and long-term applications, while native CLG is more appropriate for applications where high mechanical strength is not a primary requirement [14,44,60,66]. The choice between native and cross-linked CLG depends on the application’s requirements, including the mechanical properties, degradation rate, biocompatibility, and bioactivity.

## 3. Biomedical Properties of CLG

### 3.1. Biocompatibility and Immunogenicity

CLG’s biocompatibility has gained significant interest due to its various biomedical applications. This biocompatibility is rooted in several key characteristics, including its natural presence in the human body, its structural properties, and its ability to support cellular functions essential for tissue repair and regeneration [17]. The immune system recognizes CLG as a natural component, minimizing the risk of adverse immune responses when CLG-based biomaterials are introduced into the body. This inherent compatibility reduces the probability of rejection or inflammation, which are common issues with synthetic biomaterials [69,70]. However, the safety of CLG sourced from cattle and pigs is a significant concern due to disease risks [71]. The potential health concerns have been raised due to the incidence of bovine spongiform encephalopathy (BSE) and transmissible spongiform encephalopathy (TSE) from terrestrial animal-derived CLG and related products [72]. Allergic reactions are another critical issue. Individuals with allergies to bovine or porcine products may experience adverse reactions when exposed to CLG derived from these sources. This has been particularly noted with bovine gelatin, a common ingredient in measles, mumps, and rubella (MMR) vaccines. Some children have shown sensitivity to these vaccines, which is potentially due to the bovine CLG content [73]. Additionally, there have been reported cases of allergic reactions to bovine CLG used in medical devices, highlighting the potential for adverse immune responses in sensitive individuals [72].

Marine CLG is a potential alternative to bovine and porcine CLG due to the lower probability of disease transmission. A material obtained from fish and other aquatic organisms (i.e., marine CLG) is considered safer, which is why it is increasingly used in biomedical applications. However, the literature references show that individuals with shellfish allergies may also react adversely to marine CLG, including potential anaphylaxis [73].

The biocompatibility of CLG is also exhibited in its minimal immunogenicity. The source of CLG and the processing methods used can influence this property. Numerous studies have shown that marine CLG derived from fish (e.g., tilapia skin CLG) indicates minimal immunogenicity, making it a viable alternative to CLG of mammalian origin [74]. CLG processing methods, like electrospinning, can impact its immunogenic properties [75]. The triple helical structure, which is critical for low immunogenicity, can be preserved in self-assembled CLG nanofibers but may be altered in electrospun CLG nanofibers [76,77]. However, thorough evaluations, including cytotoxicity, hemolysis, skin sensitization, and systemic toxicity tests, confirmed the low immunogenicity and biocompatibility of CLG-based materials [78].

### 3.2. Biodegradability of CLG

CLG’s biodegradability significantly supports its application in the biomedical field. The degradation process of CLG-based materials depends on the CLG source, preparation method, and the cross-linking chemical used. Naturally derived CLG exhibits inherent biodegradability due to the presence of enzymatic and non-enzymatic pathways in the body that specifically target CLG fibers.

#### 3.2.1. Enzymatic Degradation of CLG

Enzymatic degradation is crucial to CLG’s biodegradability, especially in biomedical applications where controlled cleavage is essential for tissue remodeling and healing. Although the peptide bonds in the triple helix are occluded from enzymatic active sites, single-stranded regions are affected by matrix metalloproteinases (MMPs) [79]. Key collagenases, such as MMP-1 (collagenase-1) and MMP-8 (collagenase-2), initiate CLG degradation by cleaving its triple helical structure at specific Gly-Ile or Gly-Leu bonds, resulting in the unwinding and fragmentation of CLG fibrils into smaller peptides [80]. These fragments are further broken down by gelatinases, specifically MMP-2 and MMP-9, which target denatured CLG (gelatin) to produce even smaller peptides and amino acids [81]. The amino acids released during such an enzymatic process are utilized in various metabolic pathways that integrate CLG degradation products into normal physiological processes [80]. The activity of these enzymes is precisely regulated, maintaining tissue homeostasis and ensuring efficient turnover of the extracellular matrix (ECM) [82].

#### 3.2.2. Non-Enzymatic Degradation of CLG

Non-enzymatic degradation of CLG involves mechanisms of fiber breakdown under physiological or pathological conditions. The primary mechanism is hydrolytic degradation, where water molecules under acidic or basic conditions cleave the peptide bonds of CLG molecules [83]. Oxidative degradation, another important pathway, involves reactive oxygen species (ROS) interacting with the triple helix structure, leading to the breakdown of peptide bonds. This is particularly relevant in inflammatory conditions where ROS levels are increased [84]. Mechanical degradation also plays a role, as physical forces can cause CLG fiber fragmentation, which is often observed in load-bearing tissues subjected to repetitive stress and strain [85].

## 4. CLG-Derived Biomaterials

**Figure 3:**
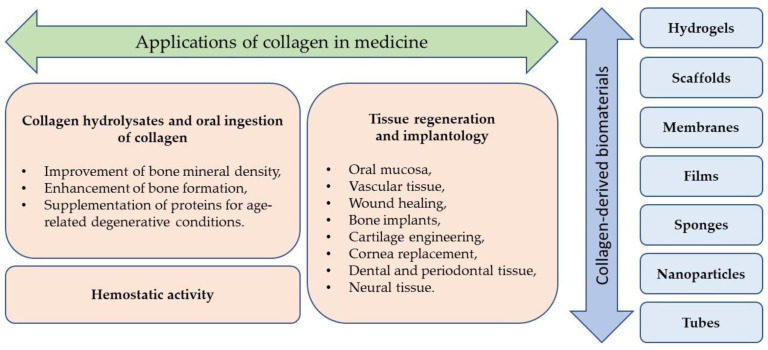
Examples of CLG-based biomaterials and their medical applications.

CLG-derived biomaterials are available in many forms and can be used in tissue engineering, regenerative medicine, and pharmacy (Table 1, Figure 3). The most common forms include membranes, scaffolds, gels, sponges, and films, and some of them are commercially available (Table 2).

### 4.1. Membranes

Membranes, usually formed as thin, flexible sheets, are used in guided bone (GBR) and tissue (GTR) regeneration. They are divided into two groups: absorbable and non-absorbable. Membranes made of polymers of natural origin (mainly CLG and chitosan) are absorbable [124]. However, a distinction can be made between cross-linked and non-cross-linked (native) CLG membranes (CLG-MB) [125,126]. They are mainly obtained from bovine or porcine tissues, such as pericardium, dermis mesenchymal tissue, or, in some cases, equine tendons. Various structures and thicknesses characterize CLG membranes. The latter does not exceed 0.5 mm. The most common forms of membranes are single-layer and two-layer materials, which consist of a layer of differently oriented CLG fibers, allowing tissue integration and a denser, permeable layer [126].

Absorbable membranes used in GBR face challenges due to their fast degradation rate, leading to early loss of barrier function, which can hinder bone formation. CLG-MBs are commonly used for regeneration and exhibit varying degradation times, with histomorphometric analysis showing a significant membrane thickness decrease within weeks to months post-surgery. Cross-linking techniques have been developed to prolong absorption time by stiffening CLG-MBs and slowing down enzymatic degradation. While cross-linked membranes are generally more resistant to degradation compared to non-cross-linked membranes, excessive cross-linking may lead to compromised healing. On the other hand, native CLG-MBs can promote bone regeneration without inflammatory or foreign body reaction only in the first 2 weeks after the surgical procedure; afterwards, they exhibit signs of degradation [127]. CLG-MBs are also used in the oral cavity, where they are especially susceptible to increased degradation due to bacterial enzymes, which affect barrier function and bone regeneration. Hence, cross-linked CLG biomaterials with enhanced membrane durability are being used for this application.

Several studies have compared the properties of cross-linked and non-cross-linked CLG membranes. Those studies have shown a correlation between the longer degradation times of cross-linked membranes; this helps in finding the right balance between the properties of such biomaterials and ensuring that they exhibit the desired requirements. The research on antibacterials and antibiotics to inhibit membrane breakdown during GBR is ongoing [96,97,128].

### 4.2. Scaffolds

A CLG scaffold (CLG-SC) is a three-dimensional structure used in tissue engineering and regenerative medicine to support the growth and repair of various types of tissues. Research has shown that the mean pore size of a scaffold is highly significant as it greatly influences the behavior of seeded cells. Scaffolds with small pore sizes (100 μm) are more effective in promoting new bone formation. Smaller pores create hypoxic conditions that stimulate cartilage formation, while larger pores (300 μm) are essential for capillary ingrowth, which is necessary for mature bone development [86].

Despite its significant quantitative presence in bone tissue and excellent biocompatibility, CLG alone does not have osteoinductive properties [129]. The combination of CLG and apatite allows such properties to be obtained [130]. Hence, the current research includes multi-component scaffolds, which, in addition to CLG, contain mainly hydroxyapatite (HAP), chitosan, or non-CLG proteins.

Various sources of CLG are being utilized in scaffold fabrication. Liu Y. et al. prepared a scaffold of human-like CLG with the addition of HAP. The results of in vitro and in vivo experiments and the relatively easy production process allowed a biomaterial to be obtained with great application potential [87]. 

A two-phase scaffold containing fish CLG (sourced from tilapia skin), HAP, and chondroitin sulphate was developed by Zhou et al. [86]. The material was composed of two layers with different pore sizes adapted to bone and cartilage regeneration, and it showed good biocompatibility and low cytotoxicity. However, it is worth mentioning that the production process did not include sterilization of the material, which may have a negative impact on the tested properties [86]. 

A scaffold of mineralized CLG with antibacterial peptides and poly(D,L-lactide-co-glycolic acid) (PLGA) was developed. The goal was to obtain a biomaterial to treat infected bone defects. The composite scaffold allowed the long-term release of the anti-inflammatory agent at the target application site for over 35 days [88].

### 4.3. Gels

CLG gels and hydrogels (CLG-G and CLG-HG) constitute a group of materials with an extensive range of current and potential medical applications. These include wound care, tissue engineering, and drug delivery systems. Tissues of porcine, bovine, or rat origin are most often used to produce CLG-Gs, but marine CLG is becoming more and more popular [131,132]. 

According to the latest edition of the International Union of Pure and Applied Chemistry (IUPAC) Gold Book, gels are described as non-fluid colloidal or polymer networks that are swollen with a fluid throughout their entire volume. Hydrogel is a gel that uses water as the swelling agent [133].

The advantages of CLG-Gs include their biocompatibility, ability to mimic the extracellular matrix, and potential for promoting tissue regeneration. The high water content in hydrogels helps to provide a moist wound environment, a crucial factor influencing the speed of wound healing. Moreover, the gel structure is flexible and adapts to the shape of the treated area without causing any discomfort to the patient. Gels can absorb excess wound exudate and remove toxins and water-soluble waste [134]. CLG-Gs also have antioxidant properties, as Shen Z. et al. demonstrated for gels obtained from fish tissues [102]. However, some disadvantages of gels may include risks related to the non-cross-linked collagen gels’ degradation products following metabolic pathways [134].

In order to obtain gels with better mechanical properties (for use as, e.g., dressing materials), additives are used in the form of synthetic polymers, e.g., polyethylene glycol, and natural polymers, such as chitosan or hyaluronic acid [135]. Various cross-linking methods are also used to obtain the desired mechanical, optical, or biological properties.

Nowadays, CLG hydrogels for injection constitute one of the research directions. Hydrogels are an excellent material for carrying the drugs and cells used in regenerative medicine (they may contain stem cells, growth factors, and drugs). Additives delivered in the hydrogel do not dissolve easily in the tissues adjacent to the application site, so their pharmacological effect is focused on the site [104]. Since cross-linked CLG is unsuitable for injection, shear-thinning, in situ cross-linking, or enzymatic cross-linking hydrogels are being developed. Those prepared using enzymatic (biological) cross-linking methods eliminate the disadvantage of toxic compounds, and according to scientific studies, cells show a high capacity for proliferation within these hydrogels, proving their excellent biocompatibility [136].

CLG-Gs can also be used to regenerate corneal damage. The challenge in creating a biomaterial for such an application, in addition to biocompatibility, is maintaining the material’s transparency and clarity. Hydrogels cross-linked by riboflavin photoactivated with UV-A light showed greater transparency than physical CLG-G [110]. Rosenquist et al. synthesized CLG chemically cross-linked with thiol. The resulting material was fully transparent, degradable by enzymes, and supported the adhesion and proliferation of human corneal cells [111]. Xeroudaki et al. [112] proposed a more complex system—a CLG hydrogel implant reinforced with nanocellulose. Chemical and physical (UV light) cross-linking methods were used, and dexamethasone was incorporated as an anti-inflammatory agent, with satisfactory drug release results (80% release within 60 days). The light transmittance of the obtained implant was comparable to the light transmittance of the human cornea [112].

### 4.4. Sponges

CLG sponges (CLG-SPs) are three-dimensional biomaterials that have been used in medicine for several decades. Due to their hemostatic properties, they are commonly used in wound treatment and dentistry (for tooth extraction). Sponges have a structure similar to a scaffold but are more flexible; they are commonly used as wound dressings and as a material for soft tissue repair.

Most commercially available CLG-SPs are of mammalian origin (bovine, equine). However, marine CLG-SPs are also currently being developed. In the work by Wang et al. [116], a CLG-SP made from tilapia skin was shown to support acute wound healing efficiently. Additional results indicated that sponge degradation products were used to create new tissue. 

A CLG-SP with the addition of taurine was developed by Wu et al. [117] as a biomaterial with antioxidant and anti-inflammatory properties. In vivo data indicated the fastest wound closure time, the best granulation process, and the best CLG deposition for the sponge among all the tested materials.

CLG-SPs can also be used in more complex systems, like a composite of CLG-SP, polydopamine, and platelet-rich plasma. It was implanted in mouse wounds and covered with a skin graft [118]. The authors indicated porosity and biocompatibility as the main advantages of the composite.

### 4.5. Films

CLG films (CLG-Fs) are thin layers often obtained by evaporating a solvent from a CLG solution. They are used in tissue engineering and are a potential material for wound treatment. Unfortunately, they have an essential disadvantage—poor mechanical properties, especially in wet environments [113]. Therefore, the current direction of research is to improve those properties. This can be done by using additives and/or cross-linking the films. Adamiak et al. examined the effect of salicin addition on the mechanical properties of CLG-F. They obtained a film with a tensile strength 50% higher than the initial material. The authors emphasized the interaction between CLG and salicin—a probable cross-linking process [113]. Tenorova et al. showed that non-cross-linked CLG-Fs with plasticizers and carboxymethylcellulose were produced as potential wound dressings. The obtained materials showed differences in mechanical properties depending on the source of CLG [114]. 

CLG-Fs, with the addition of nanoparticles, are also a research subject. An example is a film with 1% iron oxide and graphene oxide nanoparticles that significantly improved its mechanical properties (from 0.2 to 0.8 MPa), increased antioxidant activity by about 20%, and showed antibacterial properties [115].

### 4.6. Other Forms

Biomaterials in the form of microcapsules and tubes have also been developed and tested. However, such forms are less frequently studied than scaffolds, gels, or membranes. 

A CLG-based microencapsulation platform encapsulates living cells in a reconstituted nanofibrous CLG meshwork. This platform for potential osteoarthritis therapy provides a biocompatible and physiologically relevant microenvironment that supports cell attachment, proliferation, migration, and differentiation [120].

The collagen tubes (inner lumen diameter app. 0.5 mm) produced by Ishibashi et al. are biomaterials with excellent application potential. The proposed form may be used in vascular networks and nerve fibers in artificial organ fabrication and regenerative medicine technology [121]. A similar form of collagen tubes and rods with an outer diameter of 1 mm was developed by Iwamoto et al. The material’s advantages are relatively good mechanical properties (comparable to nylon) and excellent cell culture ability, which make it possible to use the material in regenerative medicine [122].

## 5. Applications of CLG in Medicine

### 5.1. CLG Hydrolysates and Oral Ingestion of CLG

CLG-derived low-molecular-weight materials are CLG hydrolysates (CLG-Hs), also known as CLG peptides. They result from breaking down CLG proteins into smaller peptide chains through hydrolysis. These peptides are rich in amino acids, like glycine, proline, and hydroxyproline, which are essential for maintaining connective tissue’s structural integrity and regeneration. The reduced size of CLG molecules enhances their absorption and bioavailability in the body. Unlike native CLG, which is insoluble in water, CLG-Hs are soluble, which makes them easier to incorporate into various formulations [137].

When taken orally, CLG-Hs offer a promising therapeutic option for managing the health issues connected with bone tissue degeneration, such as osteoarthritis [138]. Their ability to improve bone mineral density, enhance bone formation, and maintain joint and skin health underscores their potential as a multifaceted supplement for age-related degenerative conditions [139,140]. The mechanism of action for orally ingested CLG peptides involves the breaking down of CLG into smaller peptides and amino acids due to digestion in the gastrointestinal tract and their absorption into the bloodstream. Animal model studies have shown that hydroxyproline peptides could be quantified at high concentrations in human blood following the oral administration of gelatin hydrolysate, and these specific peptides can accumulate in body tissues (bones and skin) [141,142]. Wang et al. (2015), in their studies on post-cesarean section wounds in rats, proved that the oral intake of CLG peptides could improve the wound healing process [143]. 

There are examples of clinical trials implementing CLG-Hs to treat osteoarthritis and other bone-related diseases [144,145,146]. Clinical studies have demonstrated that the daily oral administration of CLG peptides can significantly increase bone mineral density in postmenopausal women [145]. In other studies, the impact of post-exercise CLG supplementation on improving the symptoms of sarcopenia has been presented [147,148]. Further research and long-term clinical trials could solidify their role in osteoporosis treatment and prevention strategies.

### 5.2. Tissue Regeneration and Implantology

Tissue regeneration techniques develop materials that restore, maintain, or improve tissue functions. This is often based on the use of a scaffold that will support the growth of new viable tissues [149]. Although many tissue regeneration materials are based on CLG of different origins, its long-term performance still lacks mechanical properties and has other limitations. For this reason, CLG is often combined/blended, and cross-linked with different additives, enriching the new composite material with the features that pure CLG lacks.

#### 5.2.1. Oral Mucosa

Oral mucosa equivalents reconstruct oral mucosa defects derived from oncologic resection, vestibuloplasty, and periodontal therapy. Terada et al. [89] developed biomaterial composed of chitosan and fish scale CLG obtained from fishery waste. Chitosan is a linear polysaccharide characterized by biodegradability, biocompatibility, low antigenicity, and bioresorbability. It is often combined with CLG to create materials for tissue regeneration. The chitosan–CLG composite scaffolds obtained in this study were seeded with oral keratinocytes from human oral mucosa samples. Those keratinocytes produced a multi-layered, polarized, stratified epithelial layer with superficial keratinization on the scaffolds, proving their potential usefulness in epithelial tissue engineering and clinical applications.

#### 5.2.2. Vascular Tissue

Wang et al. [99] used type I CLG from the scales of giant snakehead (*Channa micropeltes*) to obtain lyophilized patches. The authors performed methylation modification and 1,4-butanediol diglycidyl ether (BDE) cross-linking to improve the mechanical and degradation stability of the patches. After in vitro cytocompatibility studies, the different CLG patches were subcutaneously implanted into the hind limbs of mice, resulting in the growth of blood and lymphatic vessels around and within the patches. The authors concluded that the BDE-cross-linked methylated CLG patch promoted spontaneous lymphatic reconnection and the restoration of lymphatic flow.

#### 5.2.3. Wound Healing

Wang et al. [150] fabricated CLG sponges from tilapia skin without chemical cross-linking agents or other chemically synthesized macromolecules in order to develop medical dressings for hemostasis and wound healing. In vivo evaluation of the wound healing was performed in rats. The size of the wounds reduced significantly 7 days after CLG sponge implantation. Histological observation 21 days after wounding demonstrated that the number of capillaries in the wounds had increased significantly, with red granulation tissue gradually transforming into connective tissue with the thickening of the epidermis. In another work [90], the fish CLG/alginate (FCA) scaffold improved cell adhesion and proliferation and exhibited the best cellular compatibility in human dermal cells. The scaffold was functionalized by chitooligosaccharides (COSs) and by using 1-ethyl-3-(3-dimethylaminopropyl) carbodiimide hydrochloride as a cross-linking agent. Ge et al. [108] prepared hydrogel from pepsin-soluble CLG obtained from Nile tilapia skin. After extensive characterization of the hydrogel, the authors used it as a wound dressing to promote the healing of deep second-degree burns on rat skin. They observed faster formation of the epidermis layer and higher maturity of skin appendages for the group treated with CLG hydrogel. The outcomes suggest that the hydrogel may promote CLG proliferation and differentiation of epithelial cells.

CLG/oxidized regenerated cellulose (ORC)/silver therapy had been designed to facilitate wound healing by normalizing the microenvironment and correcting biochemical imbalances in chronic wounds [151]. The authors performed the randomized controlled trial with patients with diabetic foot ulcers for at least 30 days. As a result, they concluded that the composite CLG dressing increased healing and decreased infection levels compared with standard therapy. It was suggested that the result may be based on the ability of collagen/ORC to rebalance the chronic wound environment by reducing the high levels of protease activity that are detrimental to wound healing. 

#### 5.2.4. Bone

Due to the complex implant requirements and limited applicability of alternative devices, there is a growing need for new substitutes for bone tissue reconstruction. Natural bone tissue mainly comprises inorganic (hydroxyapatite) and organic components (CLG). Based on this, Liu et al. designed scaffolds made from nano-hydroxyapatite (n-HAP) and human-like CLG (HL-CLG). HL-CLG, expressed by recombinant *Escherichia coli* from a human mRNA sequence, has excellent water retention ability and osteoinductivity, which are useful when developing artificial bone graft substitutes. However, the insufficient mechanical strength of pure CLG scaffolds induces the addition of various additives. The n-HAP used by the authors mentioned above has the advantages of good biocompatibility, high plasticity, and remarkable mechanical properties, as its chemical and crystalline properties are analogous to bone apatite. Its ultrafine structure and high surface area are advantageous for cell–biomaterial interactions. The authors evaluated the biodegradability and histocompatibility of the scaffolds in vivo via the subcutaneous injection and bone repair effect in New Zealand rabbits as an experimental animal model. The scaffolds were implanted into the radius defects of rabbits’ legs. The materials were thoroughly degraded 12 weeks after implantation, and one of the few tested scaffolds visibly repaired the bone defect. The results showed that a new bone formed gradually as the scaffold degraded. The authors concluded that the tested materials could lead to bone regeneration, and the degradation products participated in new bone formation [87].

Double-network CLG-based hydrogels were developed to be artificially supportive tissue in implantation experiments. Swim bladder CLG and chemically cross-linked poly(N,N′-dimethylacrylamide) were used to form the double-network gels. The materials were implanted in vivo into the bone defects of rabbit knees and showed excellent biomechanical performance. Four weeks after implantation, the bonding strength of the gels was measured, showing strong osteointegration ability [109].

#### 5.2.5. Cartilage

The biomaterials used in cartilage engineering should provide mechanical support, shape, and cell-scale architecture for new tissue formation. In addition, a scaffold used for such a purpose provides the microenvironment for regenerative cell recruitment, support, proliferation, differentiation, and, finally, neo-tissue formation [94]. Due to the limited self-repair capacity of cartilage, new replacement strategies are still needed as an alternative to autologous grafts. Bertmueller et al. [92] prepared cross-linked marine CLG scaffolds for nasal cartilage repair. The scaffolds were seeded with nasal septum chondrocytes before being used in vivo. Then, the authors removed nasal septal cartilage and reconstructed that cartilage during the same procedure in rats using seeded and unseeded scaffolds. The materials did not induce any cytotoxic reactions in vitro. Chondrocytes could adhere to marine CLG and produce cartilaginous matrix proteins, such as CLG type II. Marine CLG-SCs could prevent septal perforations in an autologous, orthotopic rat model, showing excellent properties for cartilage tissue engineering [92].

Autologous chondrocyte transplantation (ACT) means the patient’s cartilage is harvested and cultivated to produce the implant. The cultivated cells are usually distributed on a three-dimensional scaffold. Once the damaged cartilage is removed, the scaffold and cells may be implanted into the articular cartilage. The study by Zak et al. [93] revealed the results 2 years after matrix-associated autologous chondrocyte transplantation using the Novocart 3D scaffold. The scaffold is a bilayered CLG type I sponge containing chondroitin sulfate, with more or less parallel plates interconnected with tiny fibers. Patients’ chondrocytes are isolated from a few small biopsy specimens arthroscopically harvested from a knee and then sent to the manufacturer, where the chondrocytes are multiplied and seeded onto the CLG-SC. Then, a patient undergoes implantation (mini-arthrotomy surgical procedure), during which the scaffold is shaped to match the exact contours of the prepared lesion. The aforementioned authors used magnetic resonance imaging for the non-invasive assessment of the status of cartilage repair tissue and its surrounding structures and early postoperative complications. After 2 years, the authors observed, in most cases, results within the range of complete filling of the lesion, between slightly incomplete filling and slight hypertrophy. Most of the transplants showed complete integration with the adjacent bone and cartilage, and in the detailed presentation of the cartilage surface, an irregularity occurred within the superior zone [93].

#### 5.2.6. Cornea

The healthy cornea is a rigid, transparent anterior eye surface essential for visual acuity [149]. CLG is a significant component of the corneal stroma that provides structural support and maintains corneal transparency. Indications for corneal transplantation include a range of diseases, from degenerative and dystrophic conditions to infectious and inflammatory corneal disorders, where the cornea, particularly the corneal stroma, loses its transparency [106]. CLG hydrogel can replace a significant portion of the corneal stroma damaged or affected by disease. CLG-based hydrogel facilitates the regeneration of the cornea by creating a framework for the migration and colonization of host cells and tissues [152]. Xeroudaki et al. [106] have recently developed a bioengineered porcine CLG (BPC) platform based on high-purity medical-grade collagen extracted from porcine skin. They used porcine CLG (type-I atelo-collagen) cross-linked by 1-[3-(Dimethylamino) propyl]77-3-ethylcarbodiimide (EDC) and N-hydroxysuccinimide (NHS) to prepare curved implants of pre-defined thicknesses, suitable for replacing a substantial portion of a damaged or diseased corneal stroma. The porous CLG hydrogel permitted migration and population by host cells while maintaining transparency and thickness six months after surgical implantation in an in vivo model of human corneal surgery in rabbits [106].

#### 5.2.7. Dental and Periodontal Tissue

The periodontium, a hierarchically organized organ comprising intercalated hard mineralized (bone and cementum) and soft unmineralized (periodontal ligament) tissues, provides mechanical and physical support to the teeth. Its regeneration remains challenging because it is difficult to reconstruct such a sophisticated mineralized/unmineralized hierarchical architecture [95]. Using biomimetic self-assembly and microstamping techniques, Yu et al. constructed a hierarchical bilayer architecture consisting of intrafibrillarly mineralized CLG resembling bone and cementum and unmineralized parallel-aligned fibrils mimicking the periodontal ligament. Those biphasic scaffolds were implanted into a complete periodontal defect model in rats. At 4 weeks after implantation, micro-CT results showed relatively continuous and intact newly formed bone in the periodontal defect area. After extending the implantation time to 8 weeks, the biphasic scaffold reconstructed a complete and functional periodontium by inserting periodontal ligament fibers into the newly formed cementum and alveolar bone. The above results indicate that the biphasic scaffolds with CLG could successfully induce stem cell multilineage differentiation into soft and hard periodontal tissues; these results are similar to the in vitro cell regulation results [95].

Allan et al. [98] developed a new CLG membrane, CelGro™, for guided bone regeneration (GBR). In GBR, bone substitute is used to fill the defect, which is then overlaid by a barrier membrane, which creates a favorable microenvironment for the repopulation of osteoprogenitor cells into bone substitute to guide bone repair and prevent non-osseous tissue ingrowth from the gingival. Bio-resorbable scaffolds are favored for GBR as they eliminate the need for post-operative retrieval. The authors proved that the developed CLG-MB acted in an osteoconductive manner to facilitate cortical bone regeneration and significantly improved cortical bone repair in the preclinical animal study. The human clinical trial involved ten participants who received GBR using CelGro™ around 16 dental implants in a two-stage clinical procedure. The trial demonstrated that GBR with CelGro™ resulted in the successful regeneration of sufficient mature bone to stabilize the dental implants and process the crown placement. The results showed satisfactory biosafety and clinical efficacy, indicating that CelGro™ CLG-MB can be used in dental and orthopedic applications [98].

#### 5.2.8. Neural Tissue

The nervous system is a crucial component of the body and restoring it following damage is a great challenge due to its complex physiology and limited regenerative capacity. CLG has been extensively studied as a biomaterial for neural tissue engineering. As a result, numerous CLG-based nerve guides are commercially available on the market for peripheral nerve regeneration [153,154]. One is NeuraGen^®^ 3D (Integra Life Sciences, Princetown, NJ, USA) —the resorbable nerve guide matrix, a product specifically engineered to create an optimized environment for peripheral nerve regeneration. Cross-linked CLG conduits are available with various inner diameters filled with a CLG–glycosaminoglycan matrix developed as an analog of the Schwann cell–ECM to create an internal framework to facilitate axonal growth [101].

### 5.3. Hemostatic Activity

An ideal hemostatic dressing should rapidly control excessive hemorrhage and be absorbable, biodegradable, non-antigenic, and biocompatible. CLG seems to fit into this description perfectly. Moreover, it can induce platelet adhesion and aggregation and release clotting factors to promote blood coagulation [155]. Additionally, CLG sponges have great sorption ability, thus promoting a hemostatic effect. Sun et al. [155] prepared CLG sponges from Nile tilapia skin by lyophilization and cross-linking with 1-ethyl-3-(3-dimethylaminopropyl) carbodiimide in the presence of N-hydroxysuccinimide (EDC/NHS). After a broad toxicity evaluation of the obtained dressings, the authors checked its hemostatic activity both in vitro and in vivo, compared with non-cross-linked sponges and commercial products. The EDC/NHS cross-linked CLG sponge showed a faster and better blood clotting ability in vitro than the commercial product. In the rat femoral artery hemorrhage model (in vivo), the cross-linked sponge exhibited the best blood clotting ability and hemostatic efficiency compared to non-cross-linked and commercial CLG sponges, exhibiting the shortest bleeding time and lowest blood loss. The work by Wang et al. [150], already mentioned in this review, evaluated CLG sponges from tilapia skin without chemical cross-linking agents or other chemically synthesized macromolecules. The hemostatic effect in vivo was measured in rats and was superior to gauze with shorter hemostasis time.

## 6. Current Research Trends

Over the years, the study of CLG has expanded beyond its traditional roles in connective tissue, revealing many functions and applications. Recently, research on CLG has witnessed a surge of innovative trends propelled by advancements in biotechnology, materials science, and medical research. This chapter delves into the cutting-edge research on CLG, highlighting the latest discoveries and their potential for use in tissue engineering, regenerative medicine, and drug delivery systems.

### 6.1. Drug Delivery Systems Based on CLG

CLG is an efficient carrier for various agents, such as genes, drugs, proteins, and growth factors. It is possible to alter CLG to fabricate materials of various durabilities, structures, and forms due to its adaptable nature [156]. Examples of such CLG-based systems are listed in Table 3. CLG nanoparticles (CLG-NPs) are more advantageous than other natural and synthetic polymeric nanoparticles as they have favorable biocompatibility and biodegradability, low antigenicity, high contact surface, reduced toxicity, and high cationic charge density potential. They are small, have a large surface area and absorptive capability, and can diffuse in water to form colloidal solutions [156,157]. They are also easily sterilized and are characterized by improved thermal stability and cell retention. Another advantage of CLG-NPs compared to native CLG is their much smaller particle size (collagen Mw is up to 300 kDa) and ability to permeate, at least partially, through the skin. Nicklas et al. [158] fabricated marine sponge CLG-NPs with estradiol-hemihydrate adsorbed to their surface for transdermal drug delivery. They applied it in a hydrogel form and compared it with a commercial gel that did not contain nanoparticles. The area under the curve (AUC) for estradiol time–concentration curves over 24 h was 2.3- to 3.4-fold higher, and the estradiol levels 24 h after administration of estradiol were at least twofold higher with the nanoparticle gel. Shalaby M. et al. presented their study results on the characterization and application of fish scale-derived CLG-NPs in wound healing [159]. Siddiqui M.A. et al. [160] presented the research results on encapsulating resveratrol in glutathione-coated CLG-NPs. This study showed that utilizing glutathione-coated CLG-NPs as DDS for resveratrol improves its effectiveness and reduces the required dose. CLG-NPs boost resveratrol’s bioavailability, while glutathione targets the brain [160]. Anwar M.M. et al. (2022) have prepared theophylline-harboring CLG-based nanoparticles as a stable and safe drug delivery system [161].

One of the advantages of drug delivery systems based on CLG is that the CLG structure is not distorted; the matrices are easily recognized by the body and contribute to the tissue repair process. CLG matrices are often used for local delivery of antibacterials, like gentamicin, providing very high local drug concentration. Such composites can be helpful in surgery, wound healing, ophthalmology, and odontology. A review by Zheng et al. [162] provides many examples of antibacterial functionalized collagen composite biomaterials. They may contain organic, inorganic, or both types of antibacterial agents. The authors conclude that such a combination forms a multi-component synergistic antibacterial system that can leverage various advantages. Hybrid usage can expand the antibacterial spectrum, providing a synergistic effect.

The PerioChip^®^ (Dexcel Pharma, Or Akiva, Israel) is an example of a second-line treatment in the care and maintenance of periodontal disease. It comes as an insert made from a biodegradable gelatin matrix containing 2.5 mg of chlorhexidine digluconate, which is released slowly for 7 days after being placed in periodontal pockets [163]. Tihan et al. [164] prepared bovine CLG sponges with ibuprofen as a potential dental delivery system. They used bovine CLG cross-linked with different amounts of glutaraldehyde to control the time of sponge degradation and drug release. The results confirmed that the higher the cross-linker concentration, the longer the sponge degradation time and the slower the drug release.

More complex drug delivery systems based on collagen are emerging, which are even more challenging to classify. The one developed by Thambirajoo et al. [165] may be an example. They prepared a sodium–carboxymethylcellulose bilayer scaffold that was later integrated with silver nanoparticles/graphene quantum dot nanoparticles as an acellular skin substitute for future use in diabetic wounds. The bilayer scaffold was prepared by layering the sodium–carboxymethylcellulose gauze onto the ovine tendon CLG type 1. The bilayer scaffold was post-cross-linked with 0.1% (*w*/*v*) genipin as a natural cross-linking agent. The cytotoxicity tests on the material demonstrated good cell viability for human epidermal keratinocytes and dermal fibroblasts.

**Table 3:** Examples of drug delivery systems based on CLG.

CLG Origin and the Type of Drug Delivery System	Drug (and Its Activity)	Route of Administration	Application	Reference
Bovine type I CLG cross-linked by glutaraldehyde, hybrid lyophilizates	irinotecan (topoisomerase I inhibitor)	transdermal	Treatment of bone and skin cancer	[166]
Bovine type I CLG cross-linked by glutaraldehyde, lyophilized sponges	niflumic acid (an analgesic and anti-inflammatory agent)	local—teeth	Pain management in dentistry and medicine	[167]
Type I CLG from calf skin, cross-linked by glutaraldehyde	lidocaine hydrochloride (local anesthetic), diclofenac sodium salt (anti-inflammatory), caffeic acid (anti-inflammatory, antioxidant)	dermal	Potential dermal application for anesthetic or anti-inflammatory action	[168]
Amphiphilic composite platform associating dense CLG hydrogels and up to 50 wt% polyesters	spironolactone (an antagonist against mineralocorticoid receptor)	not specified	Potential application in cardiovascular and renal diseases, cutaneous chronic wounds, age-related macular degeneration, chorioretinal disorders	[169]
CLG peptide and chitosan nanoparticles (pH-responsive)	doxorubicin hydrochloride (antineoplastic activity)	not specified	Significant anti-proliferative properties against HeLa (human cervical carcinoma) cells, potential innovative drug delivery carriers in advanced cancer therapy	[170]
Biomaterials made from cellulose, CLG, and polyurethane formed into thin films	ketoconazole (antifungal agent)	transcutaneous	Controlled drug release and biocidal activity	[171]
CLG (rat tail-derived) type I -hydroxyapatite scaffolds functionalized using BMP-2 and loaded with biodegradable microspheres with ALN encapsulated	bone morphogenic protein-2 (BMP-2; osteoinductive growth factor) and alendronate (ALN; treatment of bone loss and osteoporosis)	bone implantation	The initial release of BMP-2 for a few days, followed by the sequential release of ALN, after two weeks, provides enhanced bone regeneration	[172]
Ovine CLG-based micellar nanoparticles (3-ethyl carbodiimide-hydrochloride and malondialdehyde as cross-linkers)	silymarin (neuroprotective activity)	intraperitoneal	Enhanced neuroprotection by increasing drug bioavailability and targeting (in rats)	[173]
PerioChip—gelatin insert	chlorhexidine digluconate (antibacterial)	periodontal pockets	Enhanced reduction in pocket depth by approx. 0.4 mm within 6 months	[163]
Bovine CLG sponge (cross-linked with glutaraldehyde)	ibuprofen (anti-inflammatory, analgesic)	dental	Dental problems	[164]
CLG (from the marine sponge *Chondrosia reniformis*) nanoparticles	17β-estradiol-hemihydrate (hormone replacement therapy)	transdermal	Prolonged release and enhanced absorption of estradiol through human skin	[158]

### 6.2. Three-Dimensional Printing of CLG

The future of CLG-based bioinks in tissue engineering looks promising, with ongoing research focusing on improving their properties and expanding their applications. CLG and its derivatives are used to create bioinks for 3D bioprinting, which allows the construction of complex tissue structures layer by layer [174,175]. These bioinks can be combined with cells and other biomaterials to mimic tissues’ natural extracellular matrix (ECM) [176]. However, since the typically formulated bioinks based on CLG usually lack proper printability due to the slow gelation kinetics, the latest research focuses on improving the bioink’s mechanical properties. One approach to enhancing the printability and mechanical properties of CLG as a bioink is the incorporation of other synthetic and non-synthetic materials (also including cross-linking agents), such as gelatin [177], chitosan [178], alginate [175], methacrylic anhydrate [179], methacrylated hydroxybutyl chitosan [180], and many others. Also, increasing the CLG concentration in the bioink can enhance its mechanical properties. High-density CLG bioinks have been developed to improve printability and mechanical strength while maintaining cell viability [181], but high-concentration CLG can impede cell proliferation. The pH adjustment and the addition of agents like NaOH can optimize cell viability and mechanical properties [182,183].

### 6.3. CLG and Stem Cells

CLG scaffolds have gained significant attention as a supportive matrix for stem cells due to their ability to improve cell proliferation, growth, and activity [184]. Even though the first research utilizing CLG biomaterial in nerve regeneration was published over 10 years ago, there are limited examples of clinical trials on the application of CLG in stem cell therapy, specifically for spinal cord injuries. Nevertheless, new articles on this topic continue to emerge. Zou Y. et al. used an aligned CLG-SC to support a large surface area for stem cells and improve axon regeneration and remyelination [185]. In other research, CLG-SC utilization’s effects on enhancing the delivery of cells to regenerated tissue and improving stem cells’ effects in spinal injury therapy have been presented [186]. CLG-SCs provide a structural framework and significantly influence the behavior of seeded stem cells. The micro-architecture of the scaffold, including pore size and distribution, promotes the differentiation of stem cells. For instance, mesenchymal stem cells encapsulated within CLG-SCs exhibited enhanced angiogenic capacity and stemness [187].

### 6.4. Recombinant Human CLG

Animal-derived CLG presents challenges, including potential allergic reactions, disease transmission, and ethical concerns. Due to these challenges, the latest research focuses on other CLG sources and new methods of CLG synthesis, such as biotechnological methods. Recombinant human CLG (rh-CLG) represents a significant advancement in biotechnology, offering a synthetic alternative to naturally derived CLG.

Recombinant technology enables the production of human CLG by inserting human CLG genes into host cells, such as bacteria, yeast, or mammalian cells, which then produce CLG identical to that found in the human body [188,189,190]. This method ensures a purer, more consistent, and safer product, free from animal-related issues. Recombinant CLG, engineered with carefully selected or designed amino acid sequences, can achieve specific bioactivity, high purity, and consistent quality across batches. However, it also has drawbacks, including limited stability, rapid degradation, thermosensitivity, and suboptimal mechanical properties, which can be improved by mixing rhCol with additives, such as other naturally derived materials (i.e., chitosan [191], hyaluronic acid [192]) or cross-linking agents (i.e., amino acids [193,194], phosphonium salts [195]). Rh-CLG has broad applications in medicine, including tissue engineering [196,197], bone regeneration [198], and wound care [199], and holds promise for more innovative therapies and biomedical uses. RhCol can be used as a bio-mimicking material and as a substitute for natural tissues in biomedical evaluations [200,201]. Also, due to its tailored biocompatibility, rhCol is gaining interest as a bioink in tissue construction via 3D bioprinting. The information proves that rh-CLG can be successfully used as a bioink when functionalized with cross-linking agents [202,203,204].

## 7. Conclusions and Future Prospects

Understanding CLG’s structure and properties is essential to fully exploit its potential in biomaterials. CLG can exist in its native or cross-linked forms, depending on the specific application. Proper extraction conditions are crucial for obtaining materials with desired properties. 

CLG’s biocompatibility and biodegradability make it a valuable biomaterial. Due to its structure, CLG provides adequate strength and flexibility, which are essential for many tissues, such as the skin, tendons, and cartilage. Thanks to its versatility, CLG can be chemically or physically modified to obtain materials with different properties and applications. CLG-based materials can take various forms, including membranes, scaffolds, gels, sponges, films, nanoparticles, microcapsules, and tubes. The growing demand for CLG biomaterials, which is evident in the wide range of commercially available products, confirms their importance in medicine and pharmacy. Tissue regeneration and implantology are major application areas for CLG materials. These include oral mucosa, vascular, dental, periodontal, neural, bone, cartilage, and corneal tissues. CLG’s hemostatic activity makes it ideal for wound dressings. CLG hydrolysates can be used orally for various applications, including degenerative bone diseases.

Despite its numerous advantages, CLG as a biomedical material also has certain limitations. One of the primary concerns is its immunogenicity. While it is generally well tolerated by the human body, it can still elicit immune responses, mainly when derived from different species. This can lead to adverse reactions, such as inflammation, rejection, and reduced efficacy of the biomaterial. Researchers are exploring chemical modification, cross-linking, and recombinant CLG strategies to mitigate this risk. The latter, obtained using genetically engineered organisms, offers a promising alternative, as it can be produced in a controlled environment and can mitigate the risk of animal-derived pathogens.

CLG’s stability is another critical factor. It is susceptible to enzyme degradation, physical factors (like temperature and pH), and chemical agents. This can lead to loss of its mechanical properties and functional characteristics. Future research will focus on extension techniques like cross-linking and chemical modifications to enhance stability. Additionally, understanding the degradation pathways of CLG will help design biomaterials for drug delivery purposes [205].

While CLG offers suitable mechanical properties for many applications, it may not always meet the specific requirements of certain tissues or implants. For instance, in load-bearing structures like bone and cartilage, CLG may not provide sufficient strength. To address this, researchers are investigating methods to reinforce CLG with other materials or to modify its structure to improve its mechanical properties.

The production of CLG, especially in large quantities and with specific properties, can be costly. This can limit its widespread adoption in biomedical applications. Efforts are underway to develop more efficient and cost-effective production methods, including recombinant CLG production and optimization of extraction processes.

On the other hand, the commercialization of CLG-based biomaterials is restricted by numerous production regulations, particularly within the EU. Clinical trials must be preceded by a comprehensive biocompatibility assessment and stability studies, which incur substantial costs and are fraught with high risks.

Research trends are expanding CLG’s applications. One promising area is drug delivery systems, particularly CLG nanoparticles. New, eco-friendly preparation methods via biosynthesis are being developed, and their properties as nanocarriers are being evaluated. CLG-based bioinks are crucial for 3D bioprinting in tissue engineering, with research focusing on enhancing their printability and mechanical properties.

Despite the numerous applications of CLG biomaterials in medicine, certain areas could still become the focus of further research. One of these is the optimization of laboratory-scale processes in order to achieve the commercialization stage. A particularly significant challenge is the scaling up these processes and mass production, especially considering the specific requirements of proteins, which are sensitive to environmental conditions. Another critical but insufficiently studied aspect is the long-term stability and effectiveness of CLG biomaterials in vivo, which could be especially crucial in implant or prosthesis applications. Additionally, the field of oncology remains a developing area for CLG biomaterials and is awaiting further research. 

The future of CLG applications in medicine looks promising. Continued research and innovation will likely lead to more effective CLG-based materials that can accelerate tissue repair and regeneration, reduce healing time, and improve functional outcomes. This could lead to new and innovative treatment options for various medical conditions. Also, CLG biomaterials are the future of personalized medicine. The ability to tailor CLG materials to individual patients’ needs will enable more personalized and effective treatments.

## Figures and Tables

**Table 1:** Examples of CLG-derived biomaterials.

Material Form	CLG Source	Additives	Cross-Linking	In Vivo Test	In Vitro Test	Application	Result	Reference
Scaffold	Fish CLG	Chondroitin sulfate, hydroxyapatite	Yes	Yes	Yes	Osteochondral regeneration	After 6 weeks, the scaffold-treated defects were well filled with smooth, integrated tissue, unlike the empty group with irregular surfaces. By 12 weeks, the scaffold group showed complete filling with cartilage-like tissue and superior integration compared to the empty group.	[86]
Scaffold	Human-like CLG	Nano-hydroxyapatite	Yes	Yes	Yes	Bone regeneration	Twelve weeks after implantation, one of the tested scaffolds degraded completely and visibly repaired the bone defect.	[87]
Scaffold	NA	PLGA, antibacterial synthetic peptides	Yes	No	Yes	Bone regeneration	Obtaining CLG-based scaffolds with osteogenic activity and sustained release of antibacterial peptides creates an environment that promotes cell differentiation and inhibits bacteria.	[88]
Scaffold	Fish scale CLG (tilapia)	Chitosan	Yes	No	Yes	Oral mucosa therapeutic device	Oral keratinocytes from human oral mucosa produced a multi-layered, polarized, stratified epithelial layer.	[89]
Scaffold	Fish CLG (flatfish)	Chitooligosaccharides, carbodiimide derivative	Yes	No	Yes	Skin tissue regeneration	Induced cell adhesion and proliferation, promotion of well-spread cell morphology.	[90]
Scaffold	Calf skin	Hydroxyapatite, CaO fibers	Yes	Yes	Yes	Bone regeneration	Eight weeks after implantation, the condylar bone defect was wholly regenerated, and the scaffold had been completely absorbed.	[91]
Scaffold	Jellyfish	-	Yes	Yes	Yes	Nasal cartilage repair	Excellent biocompatibility with only slight evidence of local inflammatory reactions; prevention of septal perforations.	[92]
Scaffold	N/A	Chondrocytes	N/A	Yes	No	Matrix-associated autologous chondrocyte transplantation (MACT) in human knee	Partial or complete filling of the lesion in knee joint cartilage.	[93]
Scaffold	Equine	-	Yes	Yes	No	Cartilage repair	Integration into the host articular cartilage and promotion of the new cartilage-like tissue development by recruiting the host cells and driving them towards the chondrogenic differentiation, total biodegradation, and replacement of the biomaterial with the newly formed cartilage-like tissue at 16 weeks post-implantation.	[94]
Scaffold	Type I CLG	Concentrated growth factor	No	Yes	Yes	Periodontal defects healing	Eight weeks after implantation, the scaffold reconstructed a complete and functional periodontium by inserting periodontal ligament fibers into the newly formed cementum and alveolar bone.	[95]
Membrane	Porcine Peritonea	Zinc-doped nanohydroxyapatite	Yes	Yes	Yes	Guided bone regeneration	Obtained a membrane that preserved the triple helical structure of CLG fibers and their native 3D network and had a satisfactory biodegradation rate.	[96]
Membranes	Porcine (Bio-Gide^®^, Geistlich Pharma AG, Wolhusen, Switzerland), Bovine (Colla-D^®^, MedPark, Seoul, Republic of Korea)	1-ethyl-3-(3-dimethylaminopropyl) carbodiimide	Yes	Yes	Yes	Guided bone regeneration	Membranes achieved good osseointegration without cytotoxic effect, with no membrane exposure observed and no complications.	[97]
Membrane	Type I porcine CLG(CelGro™, Orthocell Ltd, Murdoch, Australia)	-	No	Yes	No	Cortical bone regeneration	CelGro™ significantly improved cortical bone repair in the preclinical animal study; in dental implant placement, GBR with CelGro™ resulted in the successful regeneration of sufficient mature bone to stabilize the dental implants and process to crown placement.	[98]
Patches	Scales of snakehead (*Channa micropeltes*)	1,4-butanediol diglycidyl ether	Yes	Yes	Yes	Subcutaneous implantation in mice	Improved cell attachment, proliferation, and infiltration of favorable growth of blood and lymphatic vessels.	[99]
CLG matrix	Porcine skin	-	No	Yes	Yes	Wound healing	The CLG matrix supports the migration of cells through the matrix, accelerating the healing process.	[100]
CLG matrix	Type I bovine	-	Yes	Yes	No	Nerve defect regeneration	Significant improvement of the nerve gap bridging and functional motor recovery in a rat model.	[101]
Gel	Fish CLG	Genipin	Yes	No	Yes	Biomaterial	Obtained CLG gels exhibit high thermal stability, antioxidant capacity, and characteristic FTIR peaks of type I CLG, indicating their potential for biomaterial applications.	[102]
Gel	Fish skin (tilapia)—CLG peptides applied in a gel form	-	No	Yes	Yes	Oral ulcer healing on dorsum tongue of mice	Healing promotion: decreased inflammatory cell infiltration, reduced TNF-αand IL-1β expression, increased fibroplasia, angiogenesis, and collagenesis trend.	[103]
Hydrogel	Type I rat tail CLG	Alginate, CaSO_4_	Yes	Yes	Yes	Cells/biomolecules delivery in surgeries	A simple method for creating pre-cross-linked injectable CLG-based hydrogels was developed, and significant cell viability results compared to similar hydrogels were achieved.	[104]
Hydrogel	Bovine	Polyethylene glycol	Yes	Yes	Yes	Corneal defects repairment	PEG-CLG hydrogels filled the defect area, remained transparent over one week, and supported multi-layered epithelial growth.	[105]
Hydrogel	Porcine	-	Yes	Yes	Yes	Corneal implantation	Positive replacement of the portion of a native corneal stroma with rapid wound healing in vivo; the implant permitted host stroma cell migration, epithelial and nerve regeneration while maintaining corneal shape and thickness during a 6-month postoperative period.	[106]
Hydrogel	Porcine skin	Chondroitin sulfate, poly-d-lysine	Yes	Yes	Yes	Wound healing	Induced fast and superior skin regeneration in a non-healing wound model in diabetic mice.	[107]
Hydrogel	Fish skin (Nile tilapia)	-	No	Yes	Yes	Healing of deep second-degree burns on rat skin	Significant acceleration of healing.	[108]
Hydrogel	Swim bladder of Bester sturgeon fish—Type I atelocollagen	Hydroxyapatite, poly(N,N′-dimethylacrylamide)	Yes	Yes	Yes	Implantation into the osteochondral defect	Four weeks after implantation, the CLG-based gel did not degrade and maintained high strength, indicating its strong osteointegration ability.	[109]
Gel	Type I bovine telocollagen	Riboflavin	Yes	No	Yes	Sealant for corneal perforation	A highly transparent gel with high adhesion between endo- and exogenous CLG was obtained.	[110]
Hydrogel	Type I porcine	DL-*N*-acetylhomocysteine thiolactone	Yes	No	Yes	Sealant for corneal perforation	Fully transparent enzyme-degradable hydrogel was obtained, and the manufacturing method allowed tuning of the gel’s mechanical properties.	[111]
Hydrogel	Type I porcine dermal CLG	Dexamethasone, 1-[3-(Dimethylamino) propyl]−3-ethylcarbodiimide me-thiodide, N-hydroxysuccinimide, riboflavin	Yes	Yes	Yes	Corneal application	A transparent CLG hydrogel reinforced by nanocellulose fibers was obtained. It can be loaded with dexamethasone to effectively reduce inflammation for at least two months post-implantation.	[112]
Film	Fish CLG (silver carp)	Salicin	Yes	No		Biomaterial application, cosmetics	The addition of salicin increases the viscosity of the solution (intermediate product) and improves the mechanical properties of CLG films.	[113]
Film	Porcine, bovine, equine	Carboxymethylcellulose, glycerine, macrogol 300	No	No		Wound treatment	Films made of equine CLG showed the highest mechanical strength and the lowest swelling ratio compared to porcine and bovine CLG.	[114]
Film	Type I marine CLG	Iron Oxide Nanoparticles, Graphene oxide Nanoparticles	No	No		Medicine, food packaging	Adding iron oxide and graphene oxide improves the films’ antioxidant, antibacterial, and mechanical properties.	[115]
Sponge	Fish, bovine, rat tail CLG	No additives	No	Yes	Yes	Wound healing	The obtained material can induce blood vessel ingrowth in the wound.	[116]
Sponge	Rat tail	Taurine	No	Yes	Yes	Wound healing	The fastest growth of the epidermis and increased level of TGF and VEGF protein secretion were observed for CLG sponges with taurine compared to CLG alone and the control sample.	[117]
Sponge	Type I bovine	Polydopamine, platelet rich plasma	Yes	Yes	Yes	Full-thickness skin defect healing	CLG sponge with polydopamine and PRP showed the highest cell adhesion and proliferation and the fastest wound healing compared to materials without polydopamine.	[118]
Sponge	Fish CLG (tilapia); bovine CLG	Polyethylene oxide, chitosan	-	Yes	No	Evaluation of wound healing in rats	Increasing the percentage of wound contraction, reducing the inflammatory infiltration, and accelerating the epithelization and healing also enhanced the total protein and hydroxyproline levels in the wound bed.	[119]
Microcapsules	Type I rat tail	Osteoarthritis chondrocytes	Yes	No	Yes	Osteoarthritis	UV-treated CLG pre-gels form hollow tubes with high stability, adjustable viscoelasticity, and controlled pore structure, which is ideal for separating endothelial and ectodermal cell cultures.	[120]
CLG tubes	Type I	Riboflavin	Yes	No	Yes	Vascular networks and nerve fibers in artificial organ fabrication and regenerative medicine	A method for producing tubes for potential biomedical applications has been developed.	[121]
Tubes/rods	Type I porcine atelocollagen	Carbonate buffer	Yes	No	Yes	Regenerative medicine	A CLG-based material with excellent mechanical properties, biocompatibility, and patentability has been developed.	[122]
Powder	Fish skin (Nile tilapia) CLG polypeptides	-	No	Yes	Yes	Evaluation of wound healing activity	High capacity to induce HaCaT cell migration; healing improvement in rabbits’ deep partial-thickness scald model.	[123]

**Table 2:** Examples of commercial collagen biomaterials.

Product/Manufacturer	Collagen Type	Additive	Material Type
Bio-Gide^®^/Geistlich Pharma	Type I, III	-	Membrane
Jason^®^/botiss biomaterials GmbH, Zossen, Germany	Type III	-	Membrane
OssixPlus^®^/Dentsply Sirona, Charlotte, NC, USA	Type I	-	Membrane
BioMend^®^/ZimVie, Westminster, CO, USA	Type I	-	Membrane
GingivAid^®^/Maxigen Biotech Inc., Taoyuan City, Taiwan	Type I	HAP, β-TCP	Scaffold
Integra Mozaik/Integra Life Sciences, Princetown, NJ, USA	Type I	TCP ^3^	Scaffold
FormaGraft/Maxigen Biotech Inc., Taoyuan City, Taiwan	Type I	HAP, TCP	Scaffold
Orthoss^®^ Collagen/Geistlich Pharma AG, Wolhusen, Switzerland	not available	Bovine HAP	Scaffold
SilvaKollagen^®^Gel/DermaRite^®^ Industries, LLC, North Bergen, NJ, USA	Type I	1% silver oxide	Gel
Woun’Dres^®^/Coloplast Corp., Minneapolis, MN, USA	not available	Panthenol, alantoine	Gel
RatuŻel/Regional Health Center Ltd., Zbąszyń, Poland	Type I	Lactic acid	Gel
Parasorb^®^/RESORBA Medical GmbH, Nürnberg, Germany	not available	-	Sponge
Hemocollagene/Septodont, Saint-Maur-des-Fossés, France	Type I	-	Sponge
Surgispon^®^/AegisLifeSciences, Ahmedabad, India	not available	-	Sponge

^1^ HAP—Hydroxyapatite; ^2^ β-TCP—β-Tricalcium Phosphate, ^3^ TCP—Tricalcium Phosphate.

## Data Availability

Not applicable.
